# Enhydrin Exhibits Antitumor Effects on Human Prostate Cancer Cells Associated with Reduced PI3K/AKT- and NF-κB-Related Gene and Protein Expression

**DOI:** 10.3390/cimb48080851

**Published:** 2026-08-21

**Authors:** Xia Zhang, Rikiya Taoka, Dage Liu, Hirohito Naito, Yohei Abe, Akram Hossain, Mikio Sugimoto

**Affiliations:** 1 Department of Urology, Faculty of Medicine, Kagawa University, 1750-1 Ikenobe, Miki-cho, Kita-gun 761-0793, Kagawa, Japan; cho.ka@kagawa-u.ac.jp (X.Z.); naito.hirohito@kagawa-u.ac.jp (H.N.); abe.yohei.1x@kagawa-u.ac.jp (Y.A.); akram.hossain@kagawa-u.ac.jp (A.H.); sugimoto.mikio@kagawa-u.ac.jp (M.S.); 2 Department of General Thoracic Surgery, Faculty of Medicine, Kagawa University, 1750-1 Ikenobe, Miki-cho, Kita-gun 761-0793, Kagawa, Japan; ryu.dagu@kagawa-u.ac.jp

**Keywords:** enhydrin, plant-derived, prostate cancer, PI3K/AKT, NF-κB, apoptosis, cell-cycle arrest

## Abstract

Enhydrin, a melampolide-type sesquiterpene lactone abundant in the leaves of yacon (*Smallanthus sonchifolius*), has not previously been investigated for its anti-prostate cancer activity. This study investigated the antiproliferative effects of enhydrin in human prostate cancer cells, its regulatory effects on apoptosis-related molecules, and its impact on the PI3K/AKT and NF-κB signaling pathways. Three prostate cancer cell lines (PC3, DU145, and LNCaP) were treated with enhydrin, and its effects were analyzed using cell viability assays, morphological observation, flow cytometry, apoptosis-focused TaqMan qPCR array, qRT-PCR, and Western blotting. In vivo antitumor activity was assessed in a PC3 xenograft mouse model. Enhydrin reduced cell viability in a dose- and time-dependent manner, induced morphological changes associated with cytotoxicity, and caused G1 cell-cycle arrest. Gene expression analysis revealed downregulation of PI3K/AKT- and NF-κB-related genes and modulation of BCL2 family genes toward a pro-apoptotic profile, which was confirmed at both mRNA and protein levels. In vivo, enhydrin suppressed tumor growth without significant body-weight loss. These findings suggest that enhydrin exerts antitumor effects in prostate cancer by reducing the expression of PI3K/AKT and NF-κB related molecules and modulating apoptosis-related proteins. Although additional studies are required to determine pathway activity, directly confirm apoptosis, and evaluate normal-cell cytotoxicity and the therapeutic window, these findings provide preliminary biological evidence of the effects of enhydrin in prostate cancer models.

## 1. Introduction

Prostate cancer is one of the most frequently diagnosed malignancies and remains a leading cause of cancer-related mortality among men worldwide [1]. Although androgen deprivation therapy (ADT) is initially effective in controlling disease progression, many patients eventually develop castration-resistant prostate cancer (CRPC), which is characterized by aggressive tumor growth, metastatic potential, and resistance to conventional therapies [2]. Therefore, the development of novel therapeutic strategies targeting key molecular pathways involved in prostate cancer progression remains an important clinical challenge.

Among the molecular pathways implicated in prostate cancer, the phosphatidylinositol 3-kinase (PI3K)/AKT signaling pathway plays a central role in regulating cell proliferation, survival, metabolism, and therapeutic resistance [3]. In addition, constitutive activation of nuclear factor-kappa B (NF-κB) has been observed in prostate cancer and is closely associated with disease progression, inflammation, and resistance to apoptosis [4]. Increasing evidence has revealed extensive crosstalk between PI3K/AKT and NF-κB signaling, contributing to enhanced tumor cell survival and therapeutic resistance [5,6]. These pathways also regulate the expression of apoptosis-related proteins, including members of the BCL-2 family, thereby promoting cancer cell survival and progression [7]. Several PI3K/AKT pathway inhibitors have entered clinical evaluation for advanced prostate cancer, although their clinical benefits remain limited because of toxicity, resistance, and pathway complexity [3,8]. Therefore, identification of novel agents capable of modulating this pathway remains an important research objective.

Natural products remain an important source of novel anticancer agents. Numerous phytochemicals exert antitumor effects through simultaneous modulation of multiple signaling pathways involved in proliferation, apoptosis, angiogenesis, and metastasis [9]. Among these compounds, sesquiterpene lactones have attracted considerable attention because of their diverse biological activities, including anti-inflammatory, antioxidant, and anticancer effects [10].

Yacon (*Smallanthus sonchifolius*), a perennial plant of the Asteraceae family, contains several bioactive sesquiterpene lactones in its leaves, including enhydrin, uvedalin, and sonchifolin [11,12]. Previous studies have shown that sesquiterpene lactones can inhibit NF-κB activation through covalent modification of critical cysteine residues within NF-κB signaling components, thereby suppressing inflammatory and survival pathways [13,14]. In addition, enhydrin and related yacon-derived sesquiterpene lactones have demonstrated antitumor activity in several cancer models, including human cervical cancer cells, through induction of caspase-dependent apoptosis and suppression of NF-κB signaling [15,16]. These findings suggest that enhydrin may act as a multi-target anticancer agent. However, its antitumor activity and underlying molecular mechanisms in prostate cancer remain unclear.

Despite remarkable advances in androgen receptor-targeted therapies, taxane chemotherapy, and immunotherapy, therapeutic resistance and disease progression remain major clinical challenges in advanced prostate cancer [17,18]. Therefore, natural products continue to attract considerable interest because they frequently modulate multiple oncogenic pathways simultaneously and may provide novel lead compounds for combination or complementary therapies [9].

Therefore, the present study aimed to investigate the antitumor effects of enhydrin in human prostate cancer cell lines and a xenograft mouse model. We further examined its effects on PI3K/AKT signaling, NF-κB-related proteins, apoptosis-associated regulators, and cell-cycle progression to clarify the molecular mechanisms underlying its anticancer activity and evaluate its potential as a therapeutic candidate for prostate cancer.

## 2. Materials and Methods

### 2.1. Cell Lines and Reagents

Human prostate cancer cell lines (DU145, PC3, and LNCaP) were obtained from the Japanese Cancer Research Bank (Tokyo, Japan). Cells were maintained in RPMI-1640 medium supplemented with 10% fetal bovine serum and 1% penicillin–streptomycin at 37 °C in a humidified incubator with 5% CO_2_. Enhydrin was kindly provided by the Applied Biological Chemistry Research Center, Kagawa University, as a white powder and was directly dissolved in the appropriate culture medium immediately before use. Because the compound was obtained from a collaborating laboratory many years ago, detailed information regarding its analytical purity is no longer available.

### 2.2. Cell Viability Assay

Cells were seeded in 96-well plates at 4 × 10^4^ cells/well, allowed to adhere for 24 h, and treated with enhydrin (0, 1.5, 3, and 4.5 μM) for 24, 48, or 72 h. Cell viability was assessed using an MTT assay (Cell Proliferation Kit I, Roche, Mannheim, Germany) following the instructions provided by the manufacturer. Absorbance was measured at 570 nm with a reference wavelength of 750 nm.

### 2.3. RNA Isolation and cDNA Synthesis

Total RNA was extracted using TRIzol reagent (Life Technologies, Carlsbad, CA, USA) and reverse-transcribed using the TaqMan Reverse Transcription Kit (Applied Biosystems, Foster City, CA, USA).

### 2.4. TaqMan Array qPCR

Gene expression profiling was performed using the TaqMan Array Human Apoptosis Fast 96-well plate (Thermo Fisher Scientific; Waltham, MA, USA, Cat. No. 4414072). The reaction mix comprised TaqMan Fast Advanced Master Mix (2×), cDNA, and nuclease-free water. Cycling conditions were set as follows: 95 °C for 20 s, followed by 45 cycles of 95 °C for 1 s and 60 °C for 20 s. Data were analyzed using DataAssist Software v3.0, using GAPDH, GUSB, and HPRT1 as endogenous controls.

### 2.5. Quantitative Real-Time PCR (qRT-PCR)

Gene-specific TaqMan probes were purchased as Gene Expression Assays (Applied Biosystems). The assay IDs were as follows: PIK3CA (Hs00907965_m1), AKT1 (Hs00178289_m1), BCL2 (Hs00608023_m1), BAK1 (Hs00832876_m1), NFKB1 (Hs00949902_m1), and GAPDH (4326317E). The qRT-PCR analyses were performed on a StepOnePlus Real-Time PCR System (Applied Biosystems); expression levels were normalized to GAPDH, and relative quantification was calculated using the comparative threshold cycle (2^−ΔΔCt^) method [19]. Relative mRNA expression levels were analyzed using DataAssist™ Software v3.0 (Applied Biosystems, Foster City, CA, USA) based on the comparative Ct method. Each assay was performed in triplicate.

### 2.6. Western Blot Analysis

For the Western blot analysis, cells were lysed in Cell Lysis Buffer M (Wako Pure Chemical Industries, Osaka, Japan). Proteins were separated using SDS-PAGE and transferred to PVDF membranes. After blocking, membranes were incubated overnight at 4 °C with primary antibodies specific to PI3CK (EP383Y; Abcam, Cambridge, UK), AKT (#4691S; Cell Signaling Technology, Danvers, MA, USA), BCL2 (ab59348; Abcam, Cambridge, UK), BAK (ab32371; Abcam, Cambridge, UK), NF-κB p65 (C22B4 Cell Signaling Technology, Danvers, MA, USA), and GAPDH (FL-335; Santa Cruz Biotechnology, Dallas, TX, USA). GAPDH was considered the loading control. Membranes were incubated with HRP-conjugated anti-rabbit IgG secondary antibody (ab6721; Abcam, Cambridge, UK) for 1 h at room temperature. Protein signals were detected using an ECL chemiluminescence kit (Amersham Biosciences, Biosciences, Amersham, UK).

### 2.7. Cell-Cycle Analysis

Cell-cycle distribution was analyzed using a Cell Cycle Analysis Kit (ab287852; Abcam). For this purpose, cells were seeded in 6 cm dishes (5000 cells/cm^2^) and allowed to adhere overnight. After treatment 24 h, cells were harvested, washed with cold PBS, and fixed by incubating them in 70% ethanol at −20 °C for ≥2 h. Fixed cells were washed using PBS and incubated in the dark with DNA staining solution containing propidium iodide and RNase A for 30 min at room temperature. DNA content was measured by flow cytometry with a minimum of 10,000 events acquired per sample. The percentages of cells in G1, S, and G2 phases were determined using appropriate analysis software.

### 2.8. Xenograft Tumor Model

We established subcutaneous xenograft tumors in 6-week-old male nude mice (BALB/cA Jcl-nu/nu; CLEA Japan, Tokyo, Japan) using PC3 cells. For this purpose, cells were subcutaneously implanted into the dorsal flank under aseptic conditions. Upon reaching a tumor volume of approximately 50 mm^3^, mice were randomly assigned to three groups (*n* = 6/group) based on treatments: vehicle control, low-dose enhydrin (0.2 mg/mouse per injection), and high-dose enhydrin (0.4 mg/mouse per injection). Enhydrin or vehicle was administered to the corresponding group via intraperitoneal injection three times weekly for 35 days. Tumor length and width were measured weekly with digital calipers, and tumor volume was calculated as (length × width^2^) × 0.5. All animal experiments were conducted following the institutional guidelines and were approved by the Animal Care and Use Committee of Kagawa University (Approval No.19637-1).

### 2.9. Statistical Analysis

Data are presented as mean ± SD based on at least three independent experiments. Differences were assessed using one-way ANOVA with Tukey’s post hoc test. *p* < 0.05 was considered statistically significant. All experiments were independently repeated at least three times.

## 3. Results

### 3.1. Enhydrin Suppresses the Viability of Prostate Cancer Cells and Alters Cellular Morphology

The antiproliferative effect of enhydrin on PC3, DU145, and LNCaP cells was assessed using an MTT assay. Cells were treated with 1.5, 3, or 4.5 μM enhydrin for 24, 48, or 72 h. Enhydrin reduced PC3 cell viability in a dose- and time-dependent manner: after 24 h of treatment, only 4.5 μM concentration induced a modest decrease, whereas all tested concentrations significantly inhibited cell viability after 48 h, with maximal inhibition at 4.5 μM. After 72 h, cell viability was further reduced across all treatment groups.

**Figure 1:**
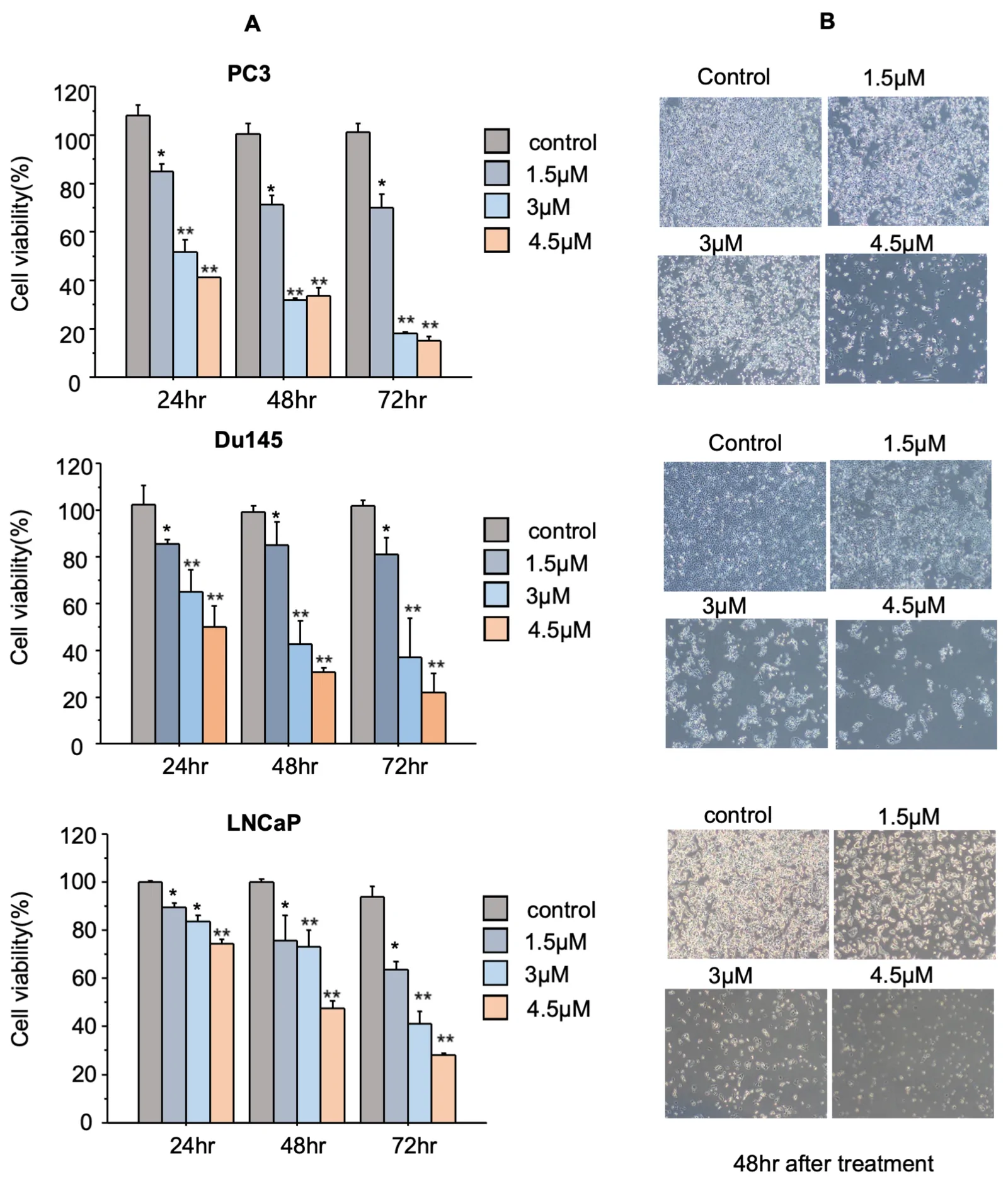
Enhydrin suppresses the viability of prostate cancer cells in a dose- and time-dependent manner. (**A**) The effects of enhydrin on cell viability were assessed by MTT assay using DU145, PC3, and LNCaP human prostate cancer cell lines. Cells were treated with 0, 1.5, 3, and 4.5 μM enhydrin for 24, 48, and 72 h. Cell viability was expressed as a percentage relative to the untreated controls. Enhydrin significantly reduced cell viability in all three cell lines in a concentration- and time-dependent manner. (**B**) Representative phase-contrast images of cells treated with the indicated concentrations of Enhydrin for 24, 48, and 72 h. Cell density decreased with increasing enhydrin concentration and prolonged exposure, accompanied by progressive morphological changes, including cell rounding, shrinkage, and detachment. Data are presented as mean ± SD of three independent experiments (n = 3). Statistical significance was determined using one-way ANOVA followed by Tukey’s post hoc test. * *p* < 0.05 and ** *p* < 0.01 versus the untreated control. Scale bar: 40 µm.

Comparable inhibitory trends were observed in DU145 and LNCaP cells, with the most pronounced suppression of viability observed at 72 h with 4.5 μM treatment (Figure 1A). Phase-contrast microscopy after 48 h of treatment revealed morphological changes consistent with cellular stress and death, including reduced cell density, rounding, shrinkage, and detachment (Figure 1B); additional original images are provided in Figure S1). These observations are consistent with the cell viability data and indicate that enhydrin exhibits cytotoxic effects on prostate cancer cells in a dose- and time-dependent manner.

### 3.2. Enhydrin Modulates Apoptosis- and Signaling-Related Gene Expression

To investigate the molecular mechanisms underlying the antitumor effects of enhydrin, we performed an apoptosis-focused TaqMan qPCR array in PC3 cells treated with 3 μM enhydrin for 48 h. Gene expression levels were normalized to GAPDH, GUSB, and HPRT1 and expressed as relative quantification (RQ) values relative to untreated controls. Enhydrin treatment significantly altered gene expression profiles, with 13 genes upregulated (RQ: 1.6–2.1) and 51 downregulated (RQ: 0.2–0.5). The complete dataset is provided in Table S1, and representative genes are summarized in Table 1, indicating a global shift toward a pro-apoptotic profile.

The complete list of differentially expressed genes identified by TaqMan qPCR array analysis is provided in Table S1; as shown in Table 1, enhydrin significantly altered genes involved in apoptosis regulation and cancer-related signaling pathways. Notably, PIK3CA, a key component of the PI3K/AKT pathway, was significantly downregulation (RQ = 0.1215). In parallel, the anti-apoptotic gene BCL2 was also reduced (RQ = 0.2488), suggesting decreased expression of molecules associated with cell survival.

Conversely, enhydrin increased the expression of pro-apoptotic genes, including BAD (RQ = 1.9804) and BAK1 (RQ = 1.9703), suggesting enhanced expression of molecules involved in intrinsic apoptosis. In addition, genes associated with NF-κB signaling were differentially regulated. The transcription factor subunit REL was downregulated (RQ = 0.2453), whereas NF-κB inhibitory molecules, including NFKBIB (RQ = 2.0060) and NFKBIE (RQ = 1.9793), were significantly upregulated. Collectively, these findings indicate that enhydrin modulates the expression of PI3K/AKT- and NF-κB-related genes together with apoptosis-related genes. This transcriptional changes may contribute to the pro-apoptotic and antiproliferative effects of enhydrin in prostate cancer cells.

### 3.3. Enhydrin Reduces the PI3K/AKT Related Gene and Protein Expression

**Figure 2:**
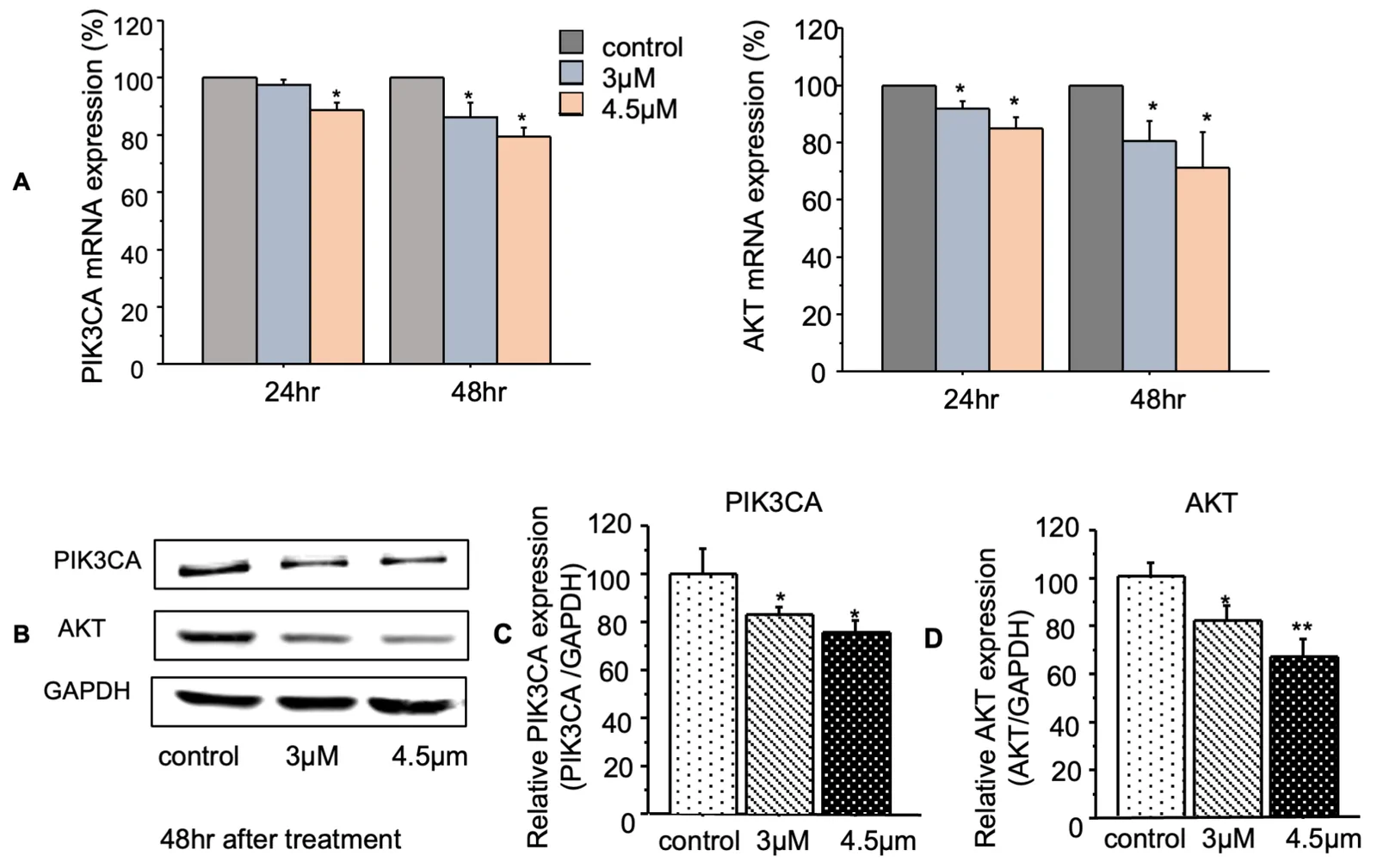
Enhydrin reduces PIK3CA and AKT expression at the mRNA and protein levels in PC3 cells. PC3 cells were treated with the indicated concentrations of enhydrin. (**A**) PIK3CA and AKT mRNA expression was determined by qRT-PCR after 24 and 48 h of Enhydrin treatment and expressed as percentages relative to the control group. (**B**) Representative Western blot images showing PIK3CA and AKT pretein expression after 48 h of enhydrin treatment, with GAPDH used as a loading control. (**C**) Densitometric quantification of PIK3CA protein expression normalized to GAPDH and expressed as relative PIK3CA expression (PIK3CA/GAPDH). (**D**) Densitometric quantification of AKT protein expression normalized to GAPDH and expressed as relative AKT expression (AKT/GAPDH). Densitometric analysis of the Western blot bands demonstrated reduced PIK3CA and AKT protein levels following Enhydrin treatment. Data are presented as mean ± SD of three independent experiments (n = 3). Statistical significance was determined using one-way ANOVA followed by Tukey’s post hoc test. * *p* < 0.05, ** *p* < 0.01 versus the untreated control.

To further investigate the effects of enhydrin on PI3K/AKT-related molecules, we analyzed key components of this pathway in PC3 cells at both the mRNA and protein levels. qRT-PCR analysis showed that enhydrin significantly reduced PIK3CA and AKT mRNA expression compared with the control group at 24 and 48 h (Figure 2A). Western blot performed after 48 h of Enhydrin treatment showed reduced PIK3CA and AKT protein levels, while GAPDH remained unchanged (Figure 2B); Full-length uncropped western blot images corresponding in Figure S2. Densitometric analysis of the Western blot bands normalized to GAPDH demonstrated a significant reduction in PIK3CA and AKT protein levels following Enhydrin treatment (Figure 2C,D). These results indicate that enhydrin reduced PIK3CA and AKT expression at both the mRNA and protein levels. Because phosphorylation status was not evaluated, these findings indicate modulation of pathway-related molecules rather than direct inhibition of PI3K/AKT signaling activity.

### 3.4. Enhydrin Regulates the Expression of BCL2 and BAK1

**Figure 3:**
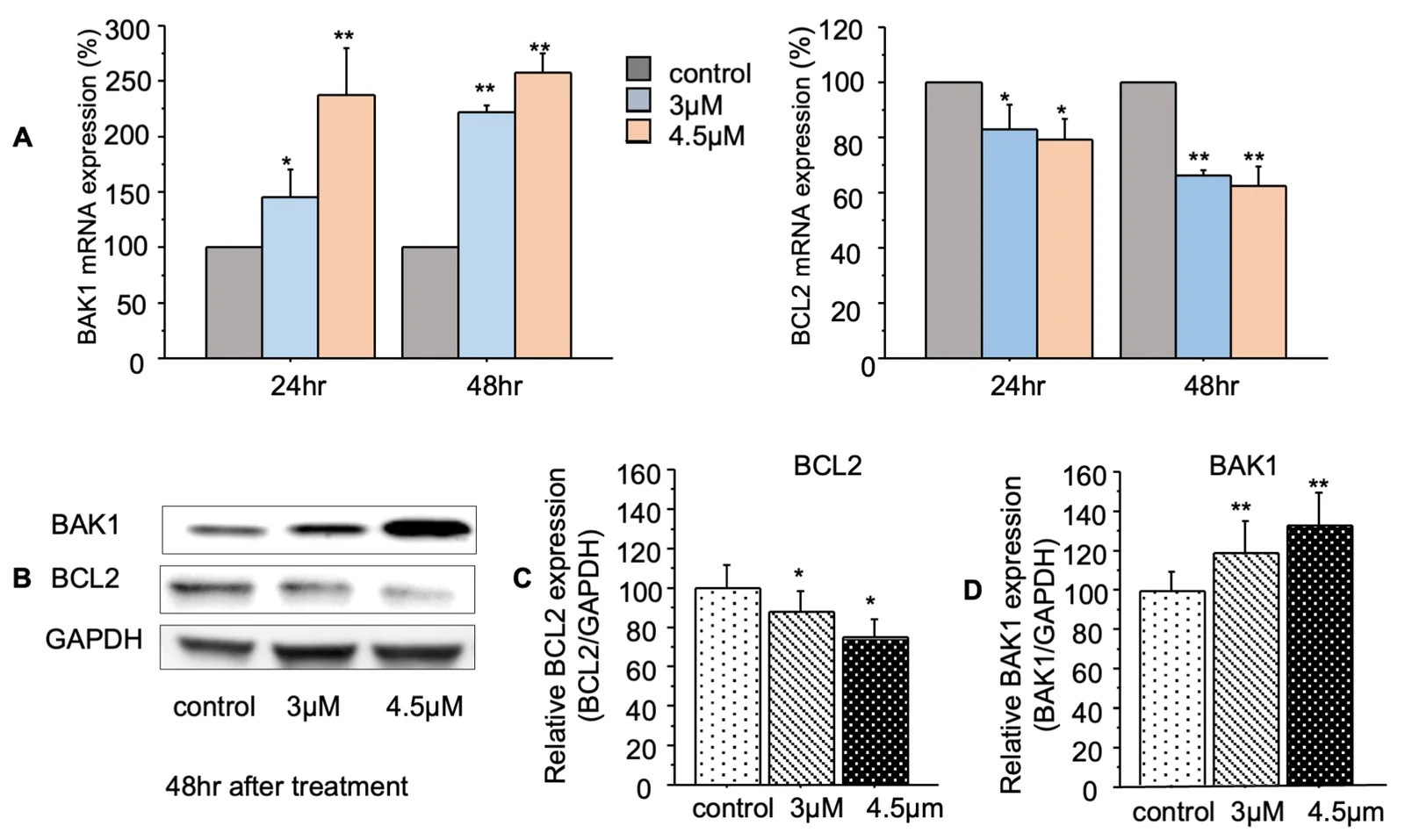
Enhydrin regulates BCL2 and BAK1 expression at the mRNA and protein levels in PC3 cells. PC3 cells were treated with the indicated concentrations of enhydrin. (**A**) BCL2 and BAK1 mRNA expression was determined by qRT-PCR after 24 and 48 h of enhydrin treatment and expressed as a percentage relative to the untreated control. (**B**) Representative Western blot images showing BCL-2 and BAK1 protein expression after 48 h of enhydrin treatment, with GAPDH used as the loading control. (**C**) Densitometric quantification of BCL2 protein expression normalized to GAPDH and expressed as relative BCL2 expression (BCL2/GAPDH). (**D**) Densitometric quantification of BAK1 protein expression normalized to GAPDH and expressed as relative BAK1 expression (BAK1/GAPDH). Densitometric analysis of the Western blot bands demonstrated a significant reduction in BCL2 protein levels and an increase in BAK1 protein levels following enhydrin treatment. Data are presented as the mean ± SD of three independent experiments (n = 3). Statistical significance was determined using one-way ANOVA followed by Tukey’s post hoc test. * p < 0.05, ** p < 0.01 versus the control group.

To assess the effects of enhydrin on mitochondrial apoptotic regulators, we analyzed the expression of BCL2 and BAK1 in PC3 cells. qRT-PCR analysis revealed that enhydrin treatment decreased BCL2 mRNA levels and increased BAK1 mRNA levels at 24 and 48 h compared with the untreated control (Figure 3A). Western blot analysis after 48 h of enhydrin treatment showed reduced BCL2 protein levels and increased BAK1 protein levels, with GAPDH used as the loading control (Figure 3B); Full-length uncropped western blot images corresponding in Figure S2. Densitometric analysis of the Western blot bands normalized to GAPDH demonstrated a significant reduction in BCL2 protein levels and an increase in BAK1 protein levels following enhydrin treatment (Figure 3C,D). BCL2 inhibits mitochondrial outer membrane permeabilization, whereas BAK1 promotes cytochrome release and caspase activation. These results indicate that enhydrin modulates the expression of BCL2 and BAK1, which may contribute to apoptosis in PC3 cells.

### 3.5. Enhydrin Reduces NF-κB-Related Gene and Protein Expression

**Figure 4:**
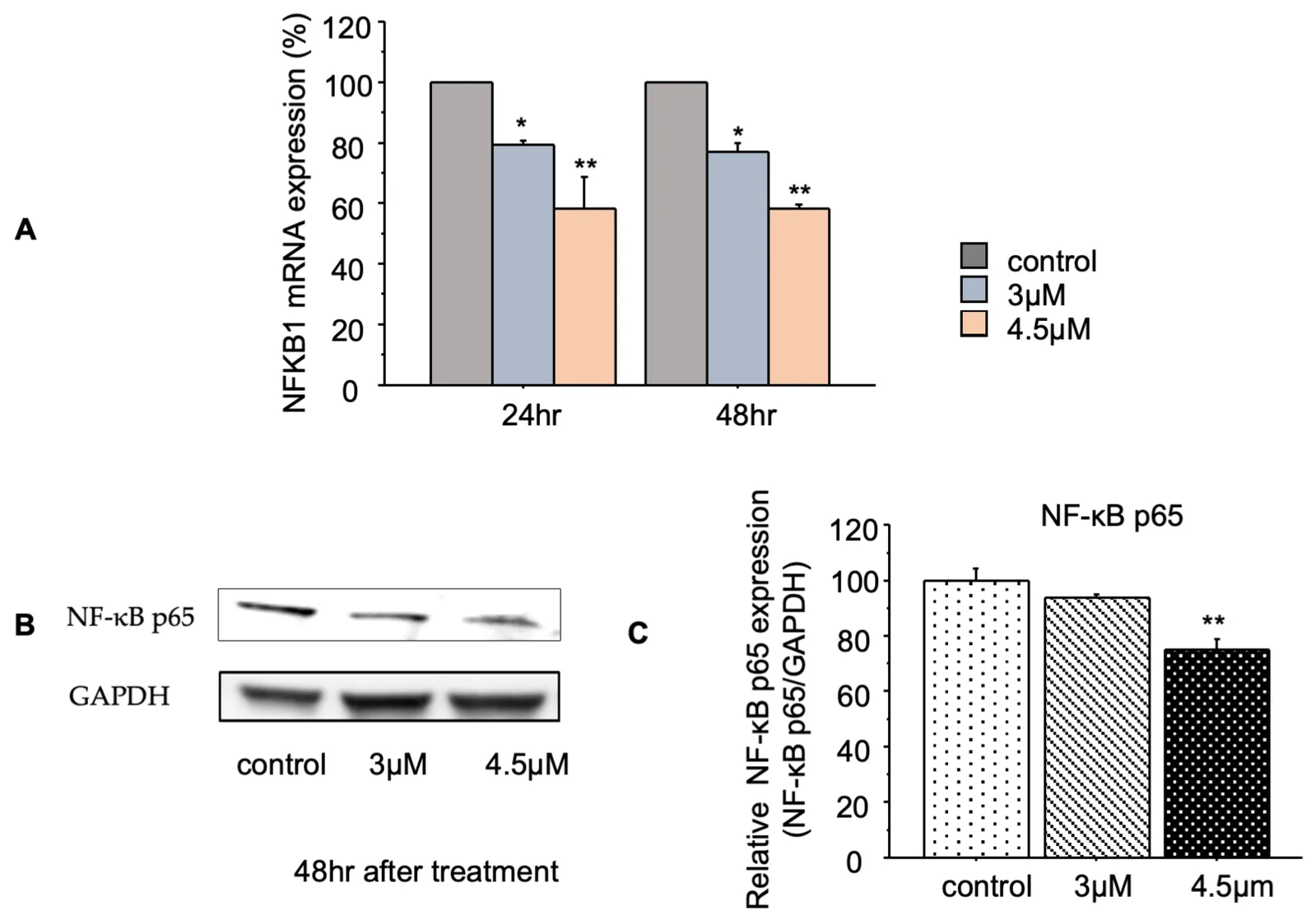
Enhydrin reduces NFKB1 mRNA and NF-κB p65 protein expression in PC3 cells. PC3 cells were treated with the indicated concentrations of enhydrin. (**A**) NFKB1 mRNA expression was determined by qRT-PCR after 24 and 48 h of enhydrin treatment and normalized to GAPDH. (**B**) Representative Western blot images showing NF-κB p65 protein expression after 48 h of enhydrin treatment, with GAPDH used as the loading control. (**C**) Densitometric quantification of NF-κB p65 protein expression normalized to GAPDH and expressed as relative NF-κB p65 expression (NF-κB p65/GAPDH). Densitometric analysis of the Western blot bands demonstrated a significant reduction in NF-κB p65 protein levels following enhydrin treatment. Data are presented as the mean ± SD of three independent experiments (n = 3). Statistical significance was determined using one-way ANOVA followed by Tukey’s post hoc test. * p < 0.05, ** p < 0.01 versus the control group.

To further investigate the effects of enhydrin on NF-κB-related molecules, we analyzed NFKB1 mRNA and NF-κB p65 protein levels in PC3 cells. qRT-PCR analysis revealed that enhydrin treatment significantly reduced NFKB1 mRNA expression at 24 and 48 h compared with the untreated control (Figure 4A). Western blot analysis after 48 h of enhydrin treatment showed decreased NF-κB p65 protein levels, while GAPDH remained unchanged (Figure 4B); Full-length uncropped western blot images corresponding in Figure S2. Densitometric analysis of the Western blot bands normalized to GAPDH demonstrated a significant reduction in NF-κB p65 protein levels following enhydrin treatment (Figure 4C). These findings are consistent with the array results, including decreased REL and increased NFKBIB/NFKBIE expression, and suggest modulation of NF-κB p65 related molecules, which may contribute to the pro-apoptotic effects of enhydrin in PC3 cells.

### 3.6. Enhydrin Alters Cell-Cycle Distribution in PC3 Cells

**Figure 5:**
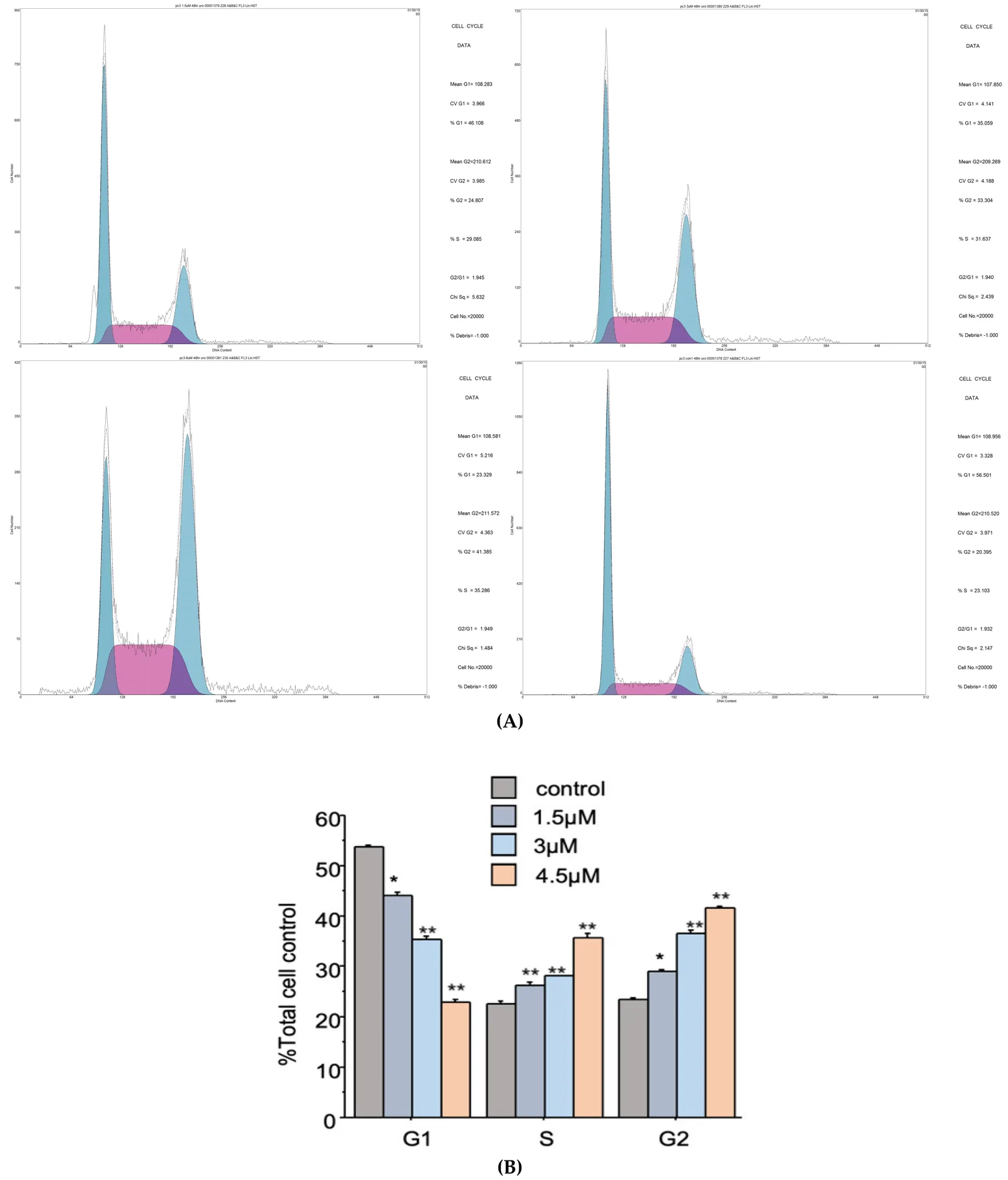
Enhydrin alters cell-cycle distribution in PC3 prostate cancer cells. PC3 cells were treated with the indicated concentrations of enhydrin for 24 h. (**A**) Representative flow cytometric histograms showing DNA content distribution in control and enhydrin-treated PC3 cells. (**B**) Quantitative analysis of the percentages of cells in the G1, S, and G2/M phases. Enhydrin treatment significantly decreased the proportion of G1-phase cells while increasing the proportions of S- and G2/M-phase cells compared with the control group. Data are presented as mean ± SD of three independent experiments (n = 3). Statistical significance was determined using one-way ANOVA followed by Tukey’s post hoc test. * *p* < 0.05, ** *p* < 0.01 versus control.

Cell-cycle distribution was analyzed by flow cytometry following treatment with enhydrin. As shown in Figure 5; Cell cycle analysis was performed using flow cytometry (Figure 5). The original raw histograms are available in Figure S3. Enhydrin significantly decreased the percentage of cells in the G1 phase, whereas the proportions of cells in the S and G2/M phases were significantly increased compared with the control group. These changes were observed in a concentration-dependent manner, indicating that enhydrin altered cell-cycle distribution in PC3 cells.

### 3.7. Enhydrin Inhibits PC3 Xenograft Tumor Growth In Vivo

**Figure 6:**
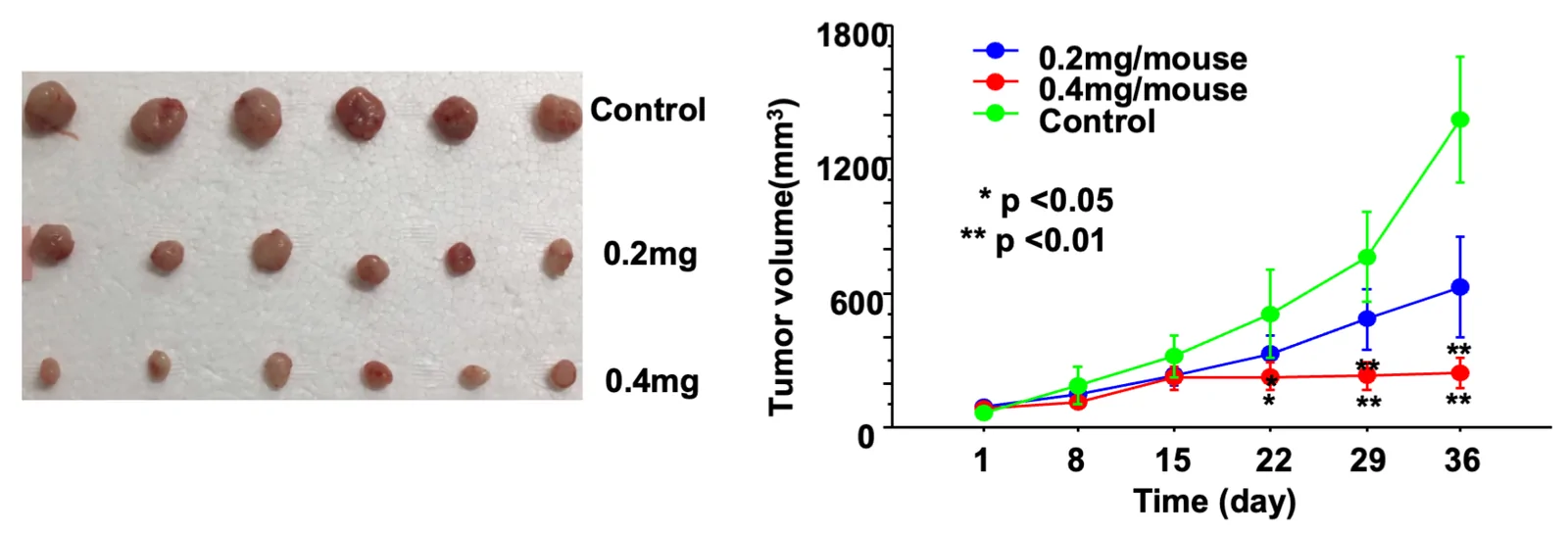
Antitumor activity of enhydrin in a PC3 xenograft mouse model. Enhydrin significantly suppressed tumor growth in PC3 tumor-bearing nude mice in a dose-dependent manner. Mice were administered phosphate-buffered saline (PBS) as a vehicle control, 0.2 mg/mouse enhydrin (low dose), or 0.4 mg/mouse enhydrin (high dose) via intraperitoneal injection three times per week. Tumor volume was measured at regular intervals using calipers and calculated as 0.5 × length × width^2^. The high-dose group exhibited the strongest inhibition of tumor growth throughout the experimental period, while the low-dose group showed a significant, but less pronounced, suppression compared with the vehicle control. Body weight was monitored, and no significant differences were observed among the three groups, under the experimental conditions used. However, the absence of significant body-weight loss does not constitute a comprehensive assessment of systemic toxicity or safety. Data are presented as mean ± SD. * *p* < 0.05, ** *p* < 0.01 versus vehicle control.

Enhydrin treatment significantly suppressed tumor growth in nude mice bearing PC3 xenografts in a dose-dependent manner compared with the vehicle control group (Figure 6). The high-dose group (0.4 mg/mouse) exhibited the strongest tumor growth inhibition, while the low-dose group (0.2 mg/mouse) also significantly inhibited tumor growth. No significant changes in body weight were observed among the treatment groups under the experimental conditions used. However, the absence of significant body-weight loss does not constitute a comprehensive assessment of systemic toxicity or safety.

## 4. Discussion

This study demonstrates that enhydrin, a melampolide-type sesquiterpene lactone derived from yacon (*Smallanthus sonchifolius*), exerts antiproliferative and pro-apoptotic effects in human prostate cancer cells and significantly suppresses tumor growth in a PC3 xenograft mouse model. Enhydrin reduced cell viability in three prostate cancer cell lines (PC3, DU145, and LNCaP) in a dose- and time-dependent manner and induced morphological changes characteristic of cytotoxicity, including cell rounding, shrinkage, and detachment. These findings extend the growing evidence that sesquiterpene lactones may function as multi-target anticancer agents and provide, to our knowledge, the first evidence of enhydrin’s activity against prostate cancer.

Further molecular analyses showed that enhydrin reduced the expression of selected PI3K/AKT and NF-κB related molecules [3,4]. These pathways play essential roles in prostate cancer progression, metastasis, and therapeutic resistance and are known to exhibit extensive crosstalk [5,6]. Therefore, simultaneous molecules associated with both pathways may offer a therapeutic advantage over agents targeting only a single pathway. Our observation that enhydrin reduced both PIK3CA and AKT1 mRNA expression and reduced PIK3CA and AKT protein levels, together with suppression of REL and induction of NF-κB inhibitors (NFKBIB and NFKBIE), suggests modulation of molecules associated with cell survival and apoptosis.

From a chemical biology perspective, sesquiterpene lactones contain electrophilic α-methylene-γ-lactone moieties capable of reacting with nucleophilic cysteine residues through Michael addition. Previous studies have shown that this mechanism contributes to NF-κB inhibition by modifying cysteine residues in p65 or IKKβ. Although we did not directly evaluate covalent adduct formation, the reduction in NF-κB p65 protein and NF-κB-related transcripts observed here is consistent with this mechanism. However, further studies are needed to determine whether enhydrin affects NF-κB nuclear translocation, DNA-binding activity, or upstream phosphorylation events such as IKK activation. Similarly, although total AKT protein levels were reduced, phosphorylation at Ser473 and Thr308 was not examined and should be investigated in future studies.

Regarding apoptosis regulation, enhydrin modulated apoptosis-related molecular markers in a manner consistent with a pro-apoptotic profile, as evidenced by decreased BCL2 and increased BAK1 and BAD expression at both mRNA and protein levels [7]. These molecular changes may be associated with mitochondrial apoptotic signaling, including mitochondrial outer membrane permeabilization and cytochrome c release, ultimately leading to caspase activation. Furthermore, upregulation of P53AIP1 and downregulation of ATM suggest additional mechanisms involving p53-mediated apoptotic sensitization and impaired DNA repair. However, apoptosis was inferred indirectly in this study, and direct evaluation of apoptotic markers such as Annexin V/PI staining, cleaved caspase-3, and PARP cleavage will be necessary to confirm this mechanism.

Enhydrin altered cell-cycle distribution, as evidenced by a decrease in the G1-phase population and an increase in the S- and G2/M-phase populations. This finding is consistent the reduced expression of PI3K/AKT-related molecules observed in the present study and suggests possible regulation of cyclins, CDKs, and CDK inhibitors such as p21 or p27, although these were not evaluated. The ability of enhydrin to simultaneously inhibit proliferation and promote apoptosis may enhance its anticancer efficacy.

In vivo, enhydrin significantly suppressed PC3 xenograft tumor growth in a dose-dependent manner without causing significant body-weight loss or overt toxicity, suggesting that it was well tolerated under the experimental conditions used in this study. However, histopathological and immunohistochemical analyses, including Ki-67, cleaved caspase-3, and TUNEL staining, were not performed and would provide important confirmation of the in vivo mechanisms. In addition, only the PC3 model was used, limiting extrapolation to other prostate cancer subtypes such as androgen receptor-positive or therapy-resistant tumors.

Our findings are consistent with previous reports showing anticancer effects of enhydrin and related yacon-derived sesquiterpene lactones in cervical cancer, lymphoma, and melanoma models, where NF-κB suppression and apoptosis induction were commonly observed. The present study extends these observations by demonstrating modulation of PI3K/AKT-related molecules in prostate cancer cells. Given the molecular heterogeneity of prostate cancer, further investigation across genetically distinct models will be important.

*Parthenolide*, *costunolide*, *alantolactone*, and *dehydrocostus lactone* are representative *sesquiterpene lactones* with well-documented anticancer activities [20,21]. These compounds have been reported to modulate multiple signaling pathways, particularly PI3K/AKT and NF-κB, thereby suppressing cell proliferation and promoting apoptosis [22]. Similar to these compounds, enhydrin reduced the expression of PI3K/AKT- and NF-κB-related molecules in the present study, suggesting that it may share common molecular characteristics with other sesquiterpene lactones. However, because pathway activity was not directly assessed, further studies are required to determine whether enhydrin exerts its antitumor effects through mechanisms comparable to those reported for these structurally related compounds.

Several limitations should be acknowledged. First, although enhydrin reduced the expression of PI3K/AKT- and NF-κB-related molecules, the activation status of these signaling pathways was not directly evaluated. Since PI3K/AKT and NF-κB signaling are primarily regulated through phosphorylation and nuclear translocation, future studies should include analyses of phosphorylated signaling proteins and related functional assays to confirm whether enhydrin directly modulates the activity of these pathways. Second, although enhydrin modulated apoptosis-related molecules, apoptosis was not directly confirmed using functional assays. Therefore, additional studies employing apoptosis-specific assays are required to validate the proposed mechanism. Third, the present study did not evaluate cytotoxicity in normal human prostate epithelial cells, Therefore, cancer-cell selectivity and a therapeutic window for enhydrin could not be established. Moreover, the absence of significant body-weight loss in the xenograft model should not be interpreted as evidence of comprehensive safety. Normal-cell cytotoxicity studies and broader toxicological evaluation are required before any translational conclusions can be drawn. Fourth, the in vivo experiments were limited to a single xenograft model, and further validation using additional models, together with comprehensive histopathological and pharmacokinetic evaluations, will be necessary to further characterize the biological effects and safety profile of enhydrin. Finally, other signaling pathways that may contribute to the antitumor effects of enhydrin were not investigated. Future studies should address these limitations and further investigate the biological effects and mechanisms of enhydrin, including its potential use in combination with current standard therapies.

## 5. Conclusions

These findings provide preliminary evidence that enhydrin reduces prostate cancer cell viability and suppresses PC3 xenograft growth under the experimental conditions used. Enhydrin also modulated the expression of PI3K/AKT- and NF-κB-related molecules and apoptosis-related proteins, although direct pathway activation and apoptosis were not functionally confirmed. Importantly, cytotoxicity in normal human prostate epithelial cells and a therapeutic window were not evaluated, and the absence of significant body-weight loss in the xenograft model does not constitute a comprehensive assessment of systemic toxicity. Therefore, further mechanistic, toxicological, and pharmacokinetic studies using additional experimental models are required before the therapeutic potential of enhydrin can be fully evaluated.

## Figures and Tables

**Table 1:** Enhydrin alters apoptosis- and signaling-related gene expression in PC3 cells.

Gene Symbol	Gene Name	Functional Category	RQ (Enhydrin vs. Control)	Regulation
PIK3CA	Phosphatidylinositol-4,5-bisphosphate 3-kinase catalytic subunit alpha	PI3K/AKT pathway	0.1215	Down
BCL2	B-cell lymphoma 2	Anti-apoptotic	0.2488	Down
BAD	BCL2-associated agonist of cell death	Pro-apoptotic	1.9804	Up
BAK1	BCL2 antagonist/killer 1	Pro-apoptotic	1.9703	Up
REL	REL proto-oncogene, NF-κB subunit	NF-κB signaling	0.2453	Down
NFKBIB	NF-κB inhibitor beta	NF-κB inhibitor	2.0060	Up
NFKBIE	NF-κB inhibitor epsilon	NF-κB inhibitor	1.9793	Up

Gene expression changes were analyzed using an apoptosis-focused TaqMan qPCR array in PC3 cells treated with 3 μM enhydrin for 48 h. Expression levels were normalized to GAPDH, GUSB, and HPRT1 and expressed as relative quantification (RQ) values relative to untreated controls. Genes are grouped according to their functional categories, including apoptosis regulators, PI3K/AKT signaling, and NF-κB-related pathways.

## Data Availability

The original contributions presented in this study are included in the article. Further inquiries can be directed to the corresponding author.

## Supplementary Materials

The following are available online at https://www.mdpi.com/article/10.3390/cimb48080851/s1.

## Abbreviations

The following abbreviations are used in this manuscript:

CRPC	Castration-resistant prostate cancer
PI3K	Phosphatidylinositol 3-kinase
RQ	Relative quantification
AKT	Protein kinase B
NF-κB	Nuclear factor-κB
PTEN	phosphatase and tensin homolog
PBS	phosphate-buffered saline
