# Supplementary material for: Enhydrin Exhibits Antitumor Effects on Human Prostate Cancer Cells Associated with Reduced PI3K/AKT- and NF-κB-Related Gene and Protein Expression
Source: Curr Issues Mol Biol. 2026 Aug 21;48(8):851. doi: 10.3390/cimb48080851 (PMC13510823; doi:10.3390/cimb48080851)
Supplement: Supplementary file 1 [file cimb-48-00851-s001.zip › cimb-4438002-Supplementary figures.pdf]

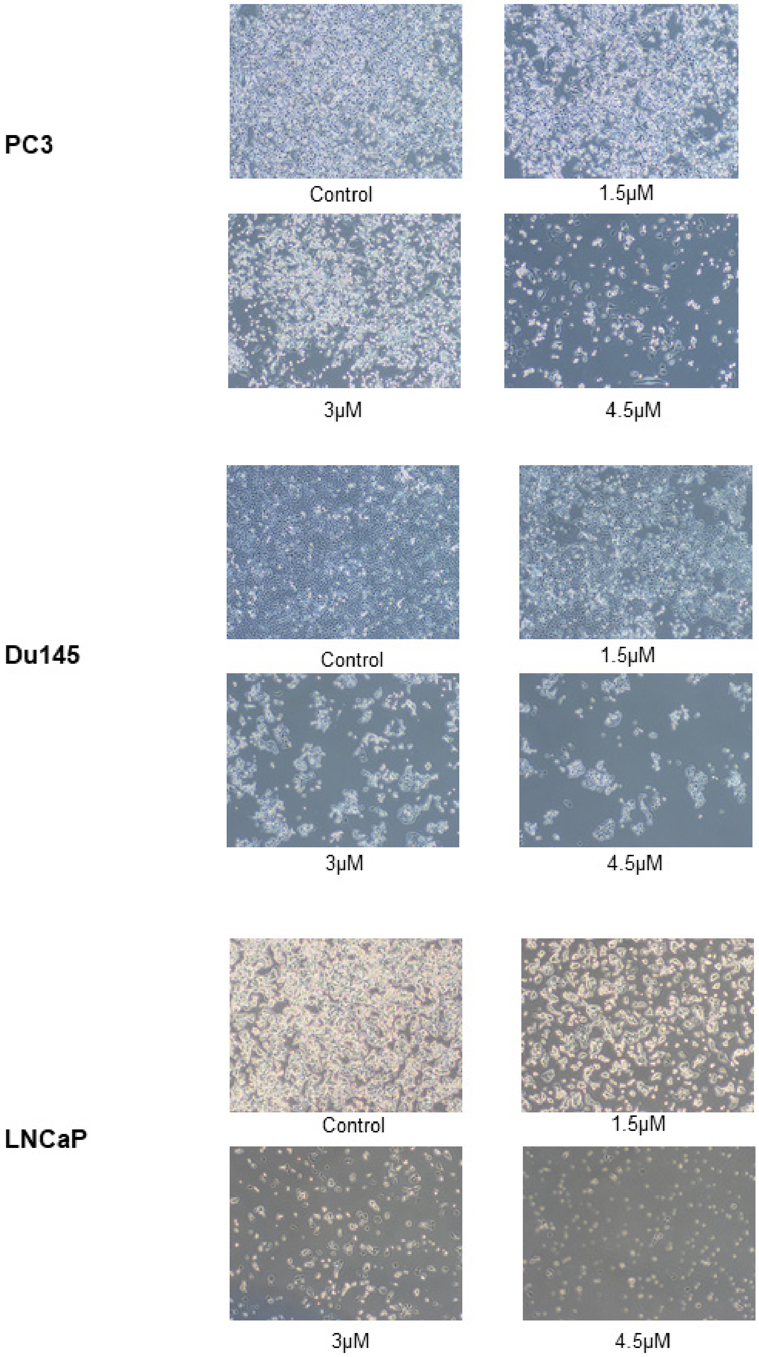

Figure S1. Original microscopic images showing the morphololgical changes of cells corresponding to Figure 1.

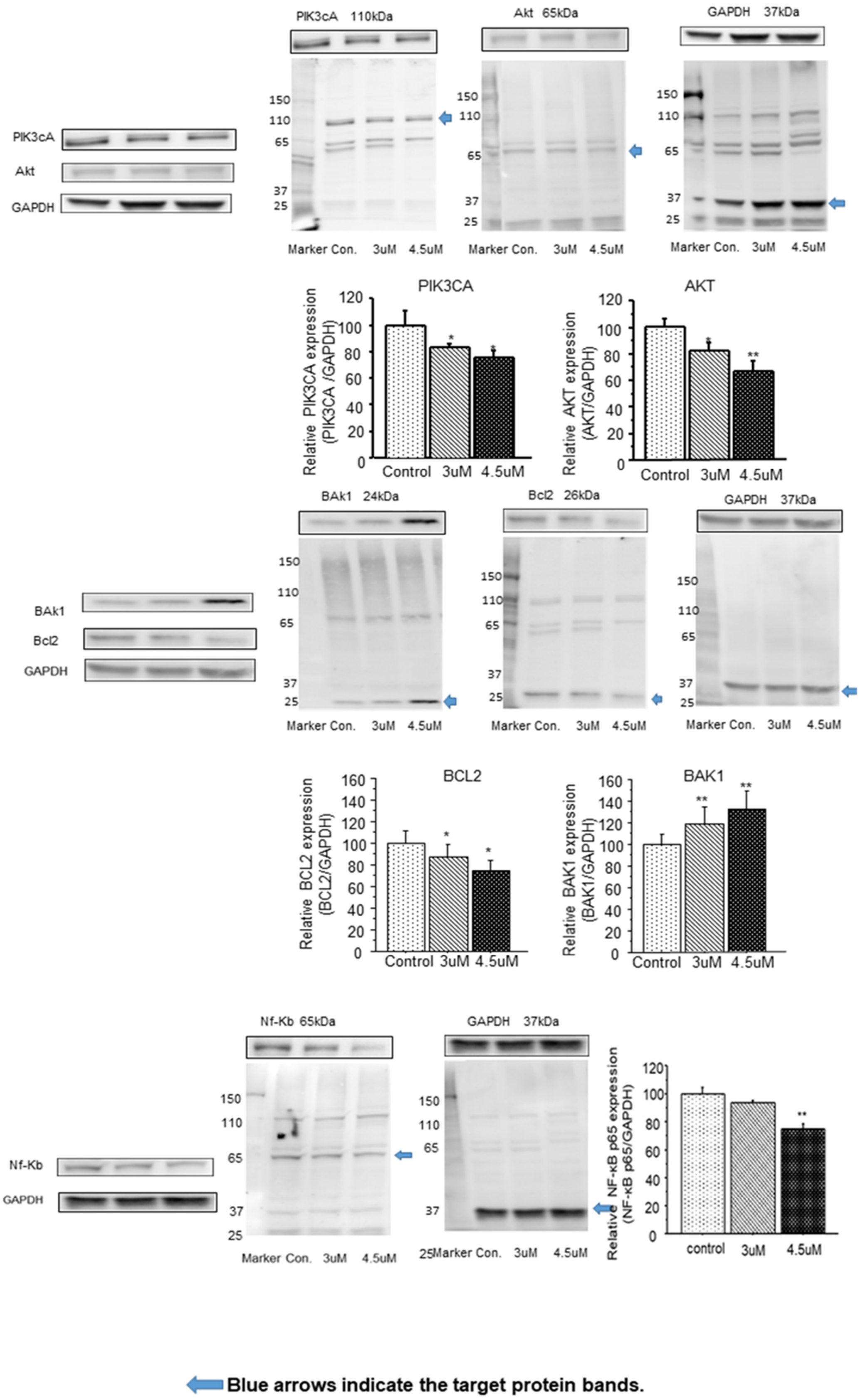

Figure S2. Full-length uncropped western blot images corresponding to Figures 2-4.

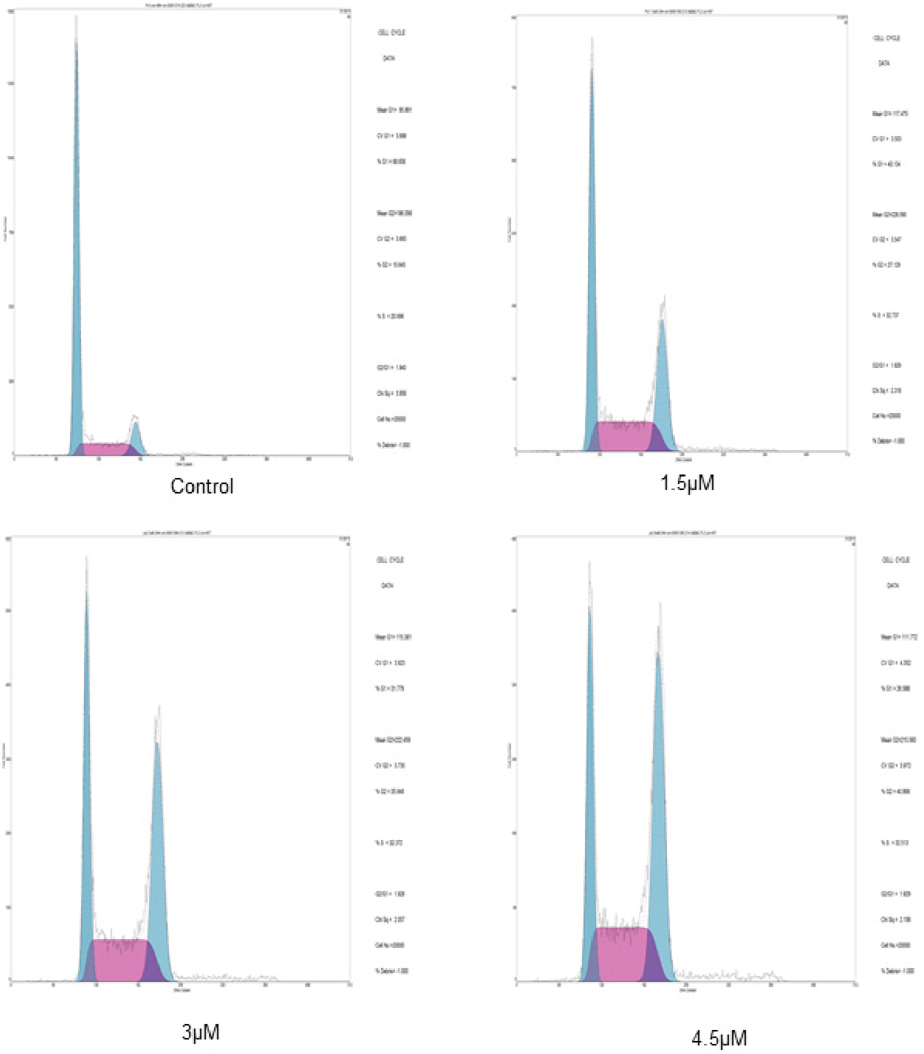

Figure S3. Original raw flow cytometry histograms corresponding to Figure 5.
